# High Mobility Group Box 1 in Human Cancer

**DOI:** 10.3390/cells9071664

**Published:** 2020-07-10

**Authors:** Bernardo L. Rapoport, Helen C. Steel, Annette J. Theron, Liezl Heyman, Teresa Smit, Yastira Ramdas, Ronald Anderson

**Affiliations:** 1 Department of Immunology, Faculty of Health Sciences, University of Pretoria, Pretoria 0001, South Africa; helen.steel@up.ac.za (H.C.S.); atheron@up.ac.za (A.J.T.); ronald.anderson@up.ac.za (R.A.); 2 The Medical Oncology Centre of Rosebank, Johannesburg 2196, South Africa; liezlheyman@gmail.com (L.H.); teresasmit@mweb.co.za (T.S.); 3 The Breast Care Centre, Netcare Milpark, 9 Guild Road, Parktown, Johannesburg 2193, South Africa; yastiraramdss@gmail.com

**Keywords:** cytokines, immunosuppression, myeloid-derived suppressor cells, prognostic factor, receptor for advanced glycation end-products, redox isoforms, Toll-like receptors, tumor microenvironment, T regulatory cells, tumorigenesis

## Abstract

High mobility group box 1 (HMGB1) is an extremely versatile protein that is located predominantly in the nucleus of quiescent eukaryotic cells, where it is critically involved in maintaining genomic structure and function. During cellular stress, however, this multifaceted, cytokine-like protein undergoes posttranslational modifications that promote its translocation to the cytosol, from where it is released extracellularly, either actively or passively, according to cell type and stressor. In the extracellular milieu, HMGB1 triggers innate inflammatory responses that may be beneficial or harmful, depending on the magnitude and duration of release of this pro-inflammatory protein at sites of tissue injury. Heightened awareness of the potentially harmful activities of HMGB1, together with a considerable body of innovative, recent research, have revealed that excessive production of HMGB1, resulting from misdirected, chronic inflammatory responses, appears to contribute to all the stages of tumorigenesis. In the setting of established cancers, the production of HMGB1 by tumor cells per se may also exacerbate inflammation-related immunosuppression. These pro-inflammatory mechanisms of HMGB1-orchestrated tumorigenesis, as well as the prognostic potential of detection of elevated expression of this protein in the tumor microenvironment, represent the major thrusts of this review.

## 1. Introduction

High mobility group box 1 (HMGB1) is a ubiquitous nuclear protein that is present in almost all eukaryotic cells, in which it plays a critical role in maintaining genomic architecture and stability [1]. It is, however, a multifaceted protein that exists in different isoforms. When released extracellularly during traumatic tissue injury of both infective and noninfective origin, HMGB1 triggers innate host defenses by acting as an alarmin [1,2,3]. If these HMGB1-activated host defense mechanisms are appropriately controlled and downregulated, they play an important protective role in the eradication of infection, as well as in promoting tissue repair [1,2,3]. However, if the release of HMGB1 by traumatized cells and tissues is excessive and prolonged, a chronic inflammatory response may occur that poses the risk of inflammation-mediated organ dysfunction, immunosuppression and tumorigenesis [1,2,3]. Increasing recognition of the involvement of HMGB1 in the pathogenesis of various types of cancer has evoked considerable awareness of the potential of this protein to serve as both a prognostic biomarker and therapeutic target in various types of human cancers [4].

The current review represents an update on the status of HMGB1 in the pathogenesis of malignant disease. The major thrusts are: (i) firstly, and most importantly, an update on HMGB1-driven pro-inflammatory mechanisms, particularly those involving interactions of HMGB1 with Toll-like receptor (TLR) 4 and the receptor for advanced glycation end products (RAGE) that promote immunosuppression and tumorigenesis in various types of cancer; and (ii) determination of the prognostic potential of in situ and systemic measurement of HMGB1 in various types of cancer. These sections are preceded by overviews of HMGB1 structure and isoforms, as well as of the role of this protein in host defense.

## 2. HMGB1 Protein Structure and Variability

**Figure 1:**
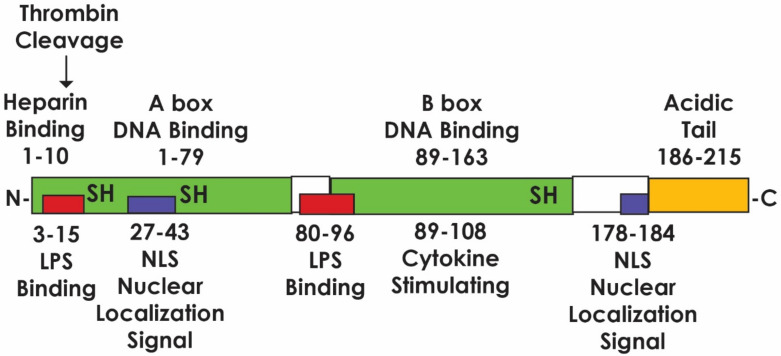
The structure of High mobility group box protein 1 (HMGB1). The A- and B-box binding moieties are shown. The three cysteines determine whether HMGB1 acts as a proinflammatory mediator when outside the cell or binds to DNA when inside the nucleus. In addition, protein stability and DNA bending in vitro is determined by the C-terminal acidic tail [15]. Adapted and reproduced from Festoff, B.W.; Citron, B.A. Thrombin and the *Coag-Inflammatory Nexus* in neurotrauma, ALS, and other neurodegenerative disorders. *Front. Neurol*. 2019. doi: 10.3389/fneur.2019.00059 under the Creative Commons Attribution 4.0 license: 4.0 license: http://creativecommons.org/license/by/4.0/.

Mammalian HMGB proteins, which are encoded by three different genes, i.e., *Hmgb1-3,* share more than 80% identity [5]. HMGB1 is expressed in almost all human cells and is released during apoptosis and necrosis, as well as by activated immune cells. The structure of the protein is presented in Figure 1. It consists of 215 amino acid residues comprising three binding domains. Two of these domains are helical deoxyribonucleic acid (DNA)-binding domains consisting of HMG A-Box (9–79 amino acid residues) and HMG B-Box (95–163 amino acid residues) [6,7,8]. The third domain comprises a shorter acidic C-terminal tail containing a series of glutamic and aspartic acid residues of various lengths (186–215 amino acid residues), which encompass RAGE and TLR binding sites [8,9,10]. HMGB1 has also been reported to bind to T-cell immunoglobulin and mucin domain 3 (TIM-3) expressed by tumor-associated dendritic cells (DCs) in murine tumors and patients with cancer [11] as one of several immunosuppressive mechanisms activated by this pleotropic protein. In addition, HMGB1 has two nuclear localization signals (NLS1 and NLS2). NLS1 has four conserved lysine residues, while five are present in NLS2. The NLS moieties serve to stabilize the chromatin structure and modulate gene transcription by bending the helical structure [12]. They are also susceptible to acetylation, resulting in exclusion of HMGB1 from the nucleus with subsequent rapid release of the protein into the cytosol [12,13,14]. The structure of HMGB1 is variable, depending on whether it is in an oxidized or reduced state (Figure 1) [15].

## 3. Oxidized and Reduced Forms of HMGB1 and Their Physiological Roles

**Figure 2:**
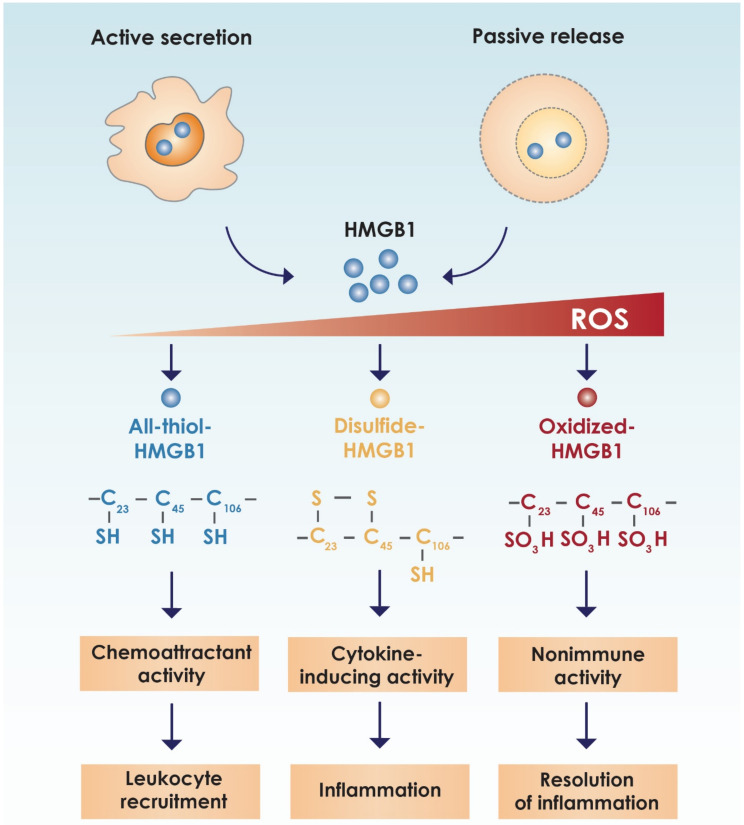
The redox state of HMGB1 determines the activity of the protein. Chemokine production and leukocyte recruitment are mediated by all-thiol-HMGB1. In turn, disulfide-HMGB1 facilitates the release of proinflammatory cytokines. During resolution of inflammation, reactive oxygen species inactivate HMGB1 by inducing the terminal oxidation of the protein [16]. Reprinted by permission from RightsLink Copyright Clearance Center: Springer Nature; Molecular Medicine. Tang, D.; Billiar, T.A.; Lotze, M.T. A Janus tale of two active HMGB1 redox states. 2012. doi:10.2119/molmed.2012.00314. License number: 4832451166310.

HMGB1 has three conserved cysteines (C) encoded at amino acid positions 23, 45 (present in A-Box) and 106 (B-Box). C23 and C45 can form an intermolecular disulfide bond, whereas C106 remains in a reduced thiol state. This allows for three different redox forms of HMGB1 namely: (i) all-thiol-HMGB1; (ii) disulfide-HMGB1; and (iii) oxidized HMGB1 [16] (Figure 2). The all-thiol isoform, with all three cysteines reduced, is the predominant type of HMGB1 in the nucleus. It is reported to be a chemokine-like molecule that forms a heterocomplex with the C-X-C motif chemokine (CXCL) 12 [stromal cell-derived factor 1 (SDF1)], thereby enhancing its chemotactic activity for monocytes via the chemokine C-X-C receptor (CXCR) 4. Furthermore, the binding of CXCL12 to CXCR4 activates the Janus kinase/signaling transducer and activator of transcription (JAK/STAT) pathway, further contributing to the recruitment of inflammatory cells to the milieu undergoing necrosis, where all-thiol-HMGB1 is also released [17]. The recruited leukocytes then produce disulfide-HMGB1 by oxidizing extracellular HMGB1 via the production of reactive oxygen species (ROS) [18].

The disulfide form of HMGB1, which is produced a few hours after all-thiol-HMGB1, activates monocytes/macrophages, as well as other cell types, to produce cytokines, chemokines and other inflammatory mediators by binding to TLR2 and TLR4. Binding of disulfide-HMGB1 to TLRs leads to the translocation of nuclear factor kappa-light-chain-enhancer of activated B cells (NFκB) to the nucleus and transcription of pro-inflammatory cytokines such as tumor necrosis factor (TNF)-α, interleukin (IL)-1, IL-6 and IL-8 [19,20]. During inflammation, disulfide-HMGB1 accumulates in the extracellular space. Terminal oxidation of HMGB1 is induced by sustained ROS production, and acts as a feedback mechanism when inflammation resolves [18]. HMGB1 enhances the activity of the transcription factors p53, p73, the retinoblastoma protein, members of the Rel/NFκB family and nuclear hormone receptors including the estrogen receptor, all of which are associated with tumor promotion [21]. Disulfide-HMGB1 and all-thiol-HMGB1 are mutually exclusive, and as such, the cytokine-stimulating and chemotactic activities of the different isoforms of HMGB1 have also been reported to be mutually exclusive [18].

Although the oxidized isoform of HMGB1 is largely thought to be noninflammatory, in that it exhibits no cytokine-stimulating or chemoattractant activities [18], Tang et al. showed that oxidized HMGB1 promotes apoptosis, mediated via the caspase-9/3 intrinsic pathway, in cancer cells [16].

## 4. Active Secretion and Passive Release of HMGB1

Under most physiological conditions, HMGB1 is localized predominantly in the cell nucleus [22]. HMGB1 contains two nonclassical nuclear export signals (NES) which, together with the two NLS sequences described above, facilitate the continuous shuttling of HMGB1 between the nucleus and the cytoplasm. During infection or cellular stress, posttranslational modifications, such as JAK/STAT-mediated acetylation, phosphorylation and methylation, result in the relocation and accumulation of HMGB1 in the cytoplasm; however, because HMGB1 lacks a leader peptide sequence, it is not actively secreted via the endoplasmic reticulum/Golgi exocytotic pathway. Consequently, in activated monocytes, HMGB1 is sequestered in secretory lysosomes, thus allowing these cells to release HMGB1 into the extracellular compartment [23,24]. Extracellular HMGB1 may act as a pro-inflammatory mediator, stimulating the release of TNF-α during infection or sterile tissue injury [25,26], as well as by promoting migration of monocytes, DCs, and neutrophils to sites of tissue injury/inflammation [27].

HMGB1 is also released passively by necrotic or damaged cells during oxidative stress [28]. The HMGB1 released by necrotic cells sends a ‘danger’ signal to neighboring cells by mediating an inflammatory response [29]. In addition to the soluble form of HMGB1, membrane microvesicles (MV) also sequester the protein [30]. These MVs are membrane protrusions released during cellular blebbing, and contain small amounts of cytoplasm. In contrast to soluble HMGB1, which is rapidly diluted in the circulation, MV-associated HMGB1 may remain in the extracellular milieu for extended periods of time and at relatively higher concentrations [31].

In the case of apoptosis, little HMGB1 is detected extracellularly, as the protein is largely retained in the nucleus from where it may be released via diffusion during the breakdown of the cellular structure [24].

## 5. Basic Function of HMGB1 in the Normal Cell: Nuclear and Cytosol Function

The function of HMGB1 is determined by its cellular location. As mentioned, HMBG1 is usually localized in the nucleus where it acts as a DNA chaperone and has an important function in maintaining DNA structure through its DNA-binding and bending activities. The HMGB1 A-Box domain is responsible for HMGB1 binding to damaged DNA, the B-Box exhibits pro-inflammatory activities, as well as DNA binding, while the C-terminal acidic tail of HMGB1 is involved in regulating DNA binding and DNA damage repair, which, in turn, confers genome stability [32,33,34]. In the nucleus, HMGB1 has been shown to be involved in replication, transcription, chromatin remodeling and V(D)J (variable, diversity and joining) recombination. In addition, HMGB1 is involved specifically in regulating the efficiency of the major DNA repair pathways. These include: (i) nucleotide excision repair (NER) [35]; (ii) base excision repair (BER) [36]; (iii) mismatch repair [37]; and iv) double-strand break repair such as nonhomologous end-joining [38]. All of these nuclear functions involve the reduced isoform of HMGB1.

With respect to its cytoplasmic location, HMGB1 is prevented from relocating to the nucleus in activated monocytes by the acetylation and phosphorylation of the protein, resulting in the accumulation of HMGB1 in the cytoplasm [39]. Cytoplasmic HMGB1 is involved in modulating cell stress responses, as well as inhibiting apoptosis via binding to, and protecting, beclin-1 and ATG5 from calpain-mediated cleavage during inflammation, while promoting autophagy and regulating mitochondrial morphology and function [40,41]. Cytoplasmic HMGB1 can either leave the cell through loss of membrane integrity or via active secretion [42].

Once released into the extracellular milieu, HMGB1 functions as a danger-activated molecular pattern (DAMP), as well as driving pro-inflammatory cytokine functions that initiate innate immune responses [43]. As mentioned above, HMGB1 also functions as a chemoattractant [44]. Extracellular HMGB1 binds to a number of pathogen-associated molecular patterns (PAMPs) which, in turn, are recognized by receptors leading to the activation of different signaling pathways, thus modulating inflammatory and immune responses, as well as promoting cell proliferation, angiogenesis, cell adhesion and migration. On the other hand, excessive accumulation of extracellular HMGB1 has been associated with the deregulation of homeostasis, promoting a wide range of acute or chronic inflammatory responses that contribute to the pathogenesis of many disorders, including diabetes, chronic sepsis, neurodegeneration, aging and cancer [2,44,45,46]. Interestingly, extracellular HMGB1 is also involved in driving the pathogenesis of several infectious diseases, including inhibition of the phagocytosis of *Pseudomonas aeruginosa* [47].

## 6. Immune Functions of HMGB1

The immune protective and suppressive functions of HMGB1 are covered briefly in this section. Apart from its nuclear and cytosolic roles as mentioned above, HMGB1 exhibits cytokine-like functions by acting as a pro-inflammatory mediator in immunity when it is secreted into the extracellular milieu. This occurs when the protein is passively released from necrotic cells, or is actively secreted by inflammatory cells such as monocytes, macrophages, natural killer cells and immature DCs, as well as platelets and endothelium following infection and exposure to inflammatory mediators [48,49,50]. Once outside the cell, HMGB1, by acting as a DAMP, mediates local or systemic immune responses via its interactions with several pattern-recognition receptors. As mentioned, these include RAGE, TLR2, TLR4, TIM-3 and CXCR4, as well as CD24-Siglec G/10 and TLR9, when combined with DNA (49). The oxidation state of HMGB1 determines its role as a chemokine or cytokine, as described below (See Figure 2) [50].

Klune et al. have described various effects of HMGB1 on cells of the innate immune system [51]. These include: (i) induction of maturation of DCs as measured by expression of surface markers and secretion of inflammatory cytokines [52,53]; (ii) an increased capacity for adhesion and transendothelial migration [54], as well as release of pro-inflammatory cytokines and other inflammatory mediators by monocytes and macrophages [55,56]; and (iii) the induction of adhesive and migratory functions of neutrophils [57] and stimulation of production of ROS through the activation of nicotinamide adenine dinucleotide phosphate (NADPH) oxidase (NOX) by these cells [58], as well as increased activation of NF-κB that results in enhanced production and release of cytokines [59]. HMGB1 has also been reported to skew macrophage polarization towards a pro-inflammatory M1-like phenotype in an experimental model of autoimmune myocarditis and systemic lupus erythematosus (SLE), and may contribute to the pathogenesis of these conditions [60,61]. Additionally, HMGB1 may mediate tumor immune escape by promoting the differentiation and proliferation, as well as the immunosuppressive activities, of myeloid-derived suppressor cells (MDSCs) [62,63].

Some of the aforementioned effects of HMGB1 on neutrophils and MDSCs are described in greater detail later in the review; the following section is focused on the effects on lymphoid cells, particularly T-lymphocytes, as well as natural killer cells and DCs.

### 6.1. HMGB1 and Dendritic Cells

Dendritic cells are professional antigen-presenting cells that effectively link the innate and adaptive arms of the immune system; they are therefore critical for the induction of protective immune responses against pathogens and tumor cells [64]. DC maturation correlates with upregulation of cell surface major histocompatibility complex (MHC) gene products, costimulatory molecules and chemokine receptors that facilitate DC migration to secondary lymphoid tissue, where these cells present antigen to T cells [65].

Several earlier studies have investigated the role of HMGB1 in modulating the maturation and function of DCs. Recombinant HMGB1, via its B-box domain, promoted the induction of phenotypic maturation of monocyte-derived DCs, as evidenced by increased expression of CD40, CD54, CD58, CD80, CD83 and MHC class II [52]. The B-box also caused enhanced secretion of the pro-inflammatory cytokines/chemokines, IL-1α, IL-6, IL-8, IL-12, TNF-α and Regulated upon Activation, Normal T Cell Expressed and Presumably Secreted (RANTES) [52]. B-box-induced secretion of IL-12 by DCs, as well as IL-2 and interferon (IFN)-γ secretion from allogeneic T cells, promoted Th1 polarization [52]. Dumitru et al., instead of using recombinant HMGB1, found that maturing DCs actively secreted HMGB1 that was responsible for autocrine maturation of these cells, which, in turn, orchestrated the priming, activation and Th1 polarization of T cells [53]. These findings suggested a role for RAGE in these events via the activation of mitogen-activated protein kinases (MAPKs) and NFκB [53].

In addition, HMGB1 affects the migratory potential of DCs, a crucial event in the accumulation of these cells in secondary lymph nodes. In one report, HMGB1 was found to act as a chemoattractant (and activator) of human DCs [66]. In another, the autocrine secretion of HMGB1 promoted remodeling of the actin-based cytoskeleton of DCs and upregulation of both the CCR4 and CXCR7 receptors [67]. The autocrine/paracrine release of HMGB1 and the integrity of the HMGB1/RAGE pathway were required for the activation of the migratory functions of DCs [67].

A very recent study by Gao et al. reported that the expression of HMBG1 was associated with the upregulation of the DC activation markers, Human Leukocyte Antigen-DR isotype (HLA-DR) and CD86, in lung cancer [68]. Further analyses revealed that HMGB1 enhanced the maturation of DCs, indicated by upregulated expression of IFN-γ in CD8^+^ T cells. HMBG1 also promoted enhanced expression of the chemokine receptors, CCR3 and CCR5, an event that resulted in an increase in DC accumulation [68]. Moreover, the resultant IFN-γ response led to elevated levels of HMGB1 and the DC-associated chemokines, C-C chemokine ligand (CCL)5, CXCL10 and CXCL11, in the tumor microenvironment (TME). The authors contend that these findings may represent an important mechanism underlying the DC-mediated antitumor immune response [68].

### 6.2. HMGB1 and Lymphoid Cells

T cells are key components of the adaptive immune system and play a critical role in orchestrating immune responses to self and foreign antigens [69]. HMGB1 has indirect effects on these cells, which, as described above, involve the induction of maturation of DCs that drives Th1 polarization [52,53]. HMGB1 also acts directly as a proliferative signal for both human CD4^+^ and CD8^+^ T cells in response to stimulation with suboptimal levels of anti-CD3 monoclonal antibody (mAb) [70].

T regulatory cells (Tregs) are a unique subset of helper T cells that suppress the immune response and appear to respond variably to HMGB1. In this context, the expression of cytotoxic T-lymphocyte-associated protein 4 (CTLA-4) and forkhead box P3 (Foxp3), proteins that are essential for the immunosuppressive functions of these cells, including IL-10 secretion, was found to be diminished following exposure to HMGB1 [71,72,73]. In contrast, another group reported that HMGB1 increased the suppressive functions of Tregs and prolonged their survival [74]. Such differences may result from variations in the techniques applied both in vitro and in experimental animal models, as evidenced by the differential signaling pathways involved (RAGE-vs TLR4-mediated signaling pathways), and possibly divergent activities of the various isoforms of HMGB1.

Some studies have supported a role for HMGB1 in B cell activation, although more research is necessary to characterize the mechanisms involved [49]. For example, it was found that when associated with DNA, HMGB1 promoted the proliferation of autoreactive B cells in response to endogenous TLR9 ligands (e.g., DNA) [14,49,75]

Natural killer (NK) cells are components of the innate immune system that show a strong cytolytic function against tumor cells and virus-infected cells [76]. HMGB1, acting in concert with other factors, promoted NK effector function and IFN-γ production following interaction with macrophages [77]. Furthermore, NK/DC crosstalk, led to the release of HMGB1 from NK cells that was pivotal for DC activation, thus favoring the onset of the adaptive immune response [78,79]. In addition, a study in transgenic mice with a targeted genomic ablation of HMGB1 in NK cells, clearly demonstrated the crucial role of this cytokine in NK development, IL-2-induced proliferation, NK cell bioenergetics and diverse NK functions, including tumor control [80].

On the other hand, HMGB1-mediated dysregulation of T cell responses, particularly those of Th17 cells that are positive regulators of the immune response, has been reported in several laboratory and clinical studies. For example, HMGB1 was found: (i) to regulate Th2 and Th17 differentiation and induction of airway inflammation in a murine model of experimental asthma [81,82]; (ii) to be elevated in the circulation of rheumatoid arthritis patients and to be positively correlated with C-reactive protein (CRP), erythrocyte sedimentation rate (ESR) and rheumatoid factor (RF), as well as with upregulation of Th17 cell activation/polarization [83,84]; and (iii) to promote progression of atherosclerosis, as well as acute allograft rejection and progression of acute graft versus host disease (GVHD), seemingly by promoting an imbalance in the Treg/Th17 ratio [85,86,87,88].

The various effects of HMGB1 on immune cells of the innate and adaptive immune systems are summarized in Table 1.

The preceding sections of this review underscore the ubiquitous cellular occurrence of HMGB1 and the increasing recognition of the role of this multifaceted protein in driving inflammatory and immune responses. Although protective, if inappropriately and chronically activated, HMGB1-driven inflammatory responses pose the potential risk of tumorigenesis, as described in the following section.

## 7. Association of HMGB1 with Cancer

HMGB1 is a somewhat enigmatic protein with respect to its role in tumorigenesis. On the one hand, intracellular HMGB1 fulfills a predominantly antitumorigenic role due to: (i) the maintenance of genome structure and stability [1,89] which, in turn, may restrict the potential for mutational diversity of genes that encode tumor antigens, thereby preventing evasion of immune recognition by tumor-infiltrating lymphocytes (TILs); and (ii) through its extracellular release during the process of immunogenic cell death (ICD), HMGB1 has been implicated in driving tumor cell death via the activation of innate and adaptive antitumor immune responses as described above [90,91,92,93,94].

On the other hand, high-level expression of HMGB1 is evident in the microenvironments of many types of advanced malignancies, originating from tumor cells per se, and from tumor-infiltrating myeloid cells in particular, as well as lymphoid cells [95] and structural cells such as endothelial cells [96] and fibroblasts [97]. In the setting of the TME, HMGB1 has been implicated in the pathogenesis of various stages of tumorigenesis, including promotion, progression and spread [98].

Malignancies in which HMGB1 has been reported to play a prominent role in disease pathogenesis, albeit often involving variable mechanisms operating at different stages of tumorigenesis, include non-small cell lung cancer (NSCLC) [99], metastatic pancreatic ductal adenocarcinoma [100,101], metastatic breast cancer [21,102], epithelial ovarian cancer [103], hepatocellular carcinoma (HCC) [104,105], colorectal cancer [106,107], metastatic melanoma [108], esophageal squamous cell carcinoma [109], malignant mesothelioma [110] and glioblastoma [111].

## 8. HMGB1 and Tumorigenesis

The following sections of the review are focused on putative mechanisms that underpin the involvement of HMGB1 in tumor promotion, progression and invasion/metastasis.

### 8.1. HMGB1 in Tumor Promotion

Several mechanisms may contribute to the pathogenesis of HMGB1-associated tumor promotion, most prominent among which is an indirect mechanism linked to perpetuation of chronic inflammation of both infective and noninfective origin [50]. In these settings, the activation of vascular endothelium [112,113,114], platelets [115], tissue macrophages [43,116] and possibly parenchymal cells [117] at sites of tissue injury results in both the active and passive release of HMGB1 from these cells. Platelets, although anucleate cells, represent a major source of HMGB1 that is acquired from megakaryocytes during thrombopoiesis and is located in the cytosol of these cells, as well as in platelet vesicles, as the unmodified protein or its encoding mRNA [115,118].

Depending on the nature of the cellular insult and the cell type, mechanisms of release of HMGB1 include: (i) the activation of the transcription factor, (NFκB) [112]; (ii) the activation of nucleotide-binding oligomerization domain (NOD)-like receptor family pyrin domain containing protein three (NLRP3) inflammasomes [43,114,116], which, in the case of macrophages, appears to involve triggering of the enzyme double-stranded RNA-dependent protein kinase [43]; and (iii) the intracellular generation of ROS (of mitochondrial origin or generated via activation of NOX family enzymes), specifically hydrogen peroxide (H_2_O_2_); this, in turn, results in Ca^2+^ overload and activation of the Ca^2+^-dependent enzymes, protein kinase Cα (PKCα) and Ca^2+^/calmodulin-dependent protein kinase IV (CaMKIV) [117]. These enzymes then mediate the cytosolic translocation and posttranslational phosphorylative modification of nuclear HMGB1 [117].

In the extracellular environment, HMGB1, as mentioned, exists as two distinct pro-inflammatory variants according to differential redox modification of the three conserved cysteine residues, i.e., C23, C45 and C106. Firstly, the isoform in which all three cysteines remain in the reduced form [18,119,120] possesses chemotactic activity that is dependent on the formation of a heterocomplex with the chemokine, CXCL12 [121,122,123,124]. This interaction with HMGB1 augments the affinity of the chemokine for its receptor, CXCR4, expressed on various types of immune/inflammatory cells, including monocytes/macrophages and T lymphocytes [121,122,123,124]. The augmentation of the intensity of CXCR4-driven intracellular signaling mechanisms following interaction with the HMGB1/CXCL12 heterocomplex, potentiates the recruitment of inflammatory cells to sites of tissue injury [121,122,123,124].

The second pro-inflammatory isoform of HMGB1 results from the selective oxidative modification of the protein, specifically, the oxidation of the proximal cysteine residues, C23 and C45, with the resultant formation of an intramolecular disulfide bond with retention of C106 in the unmodified (reduced) state [19]. Both of these events are essential in conferring pro-inflammatory activity on this variant of HMGB1 via its interactions with TLR4, a pathogen recognition receptor (PRR) broadly expressed on various types of structural cells and immune/inflammatory cells, particularly those of the innate immune system linked to the production of pro-inflammatory cytokines, including TNF-α [19].

With respect to activation of RAGE, also expressed on various cell types, including inflammatory cells and tumor cells [1], earlier studies identified fully reduced HMGB1 as the most prominent ligand of the two HMGB1 isoforms for this ubiquitous pro-inflammatory/pro-oxidative receptor [125]. However, more recent studies have contended that the relative potencies of the HMGB1 isoforms as ligands for RAGE remain to be conclusively established [126].

Importantly, the involvement of the prominent pro-inflammatory isoforms of HMGB1, together with that of other contributory mechanisms, in initiating and sustaining harmful, chronic inflammatory responses poses a well-recognized and ominous risk for the development of epithelial cancers at sites of sustained inflammation-mediated tissue injury [127]. With respect to tumor promotion, ROS, specifically H_2_O_2_ and hydroxyl radicals, generated by infiltrating phagocytic cells, are potent carcinogens [128]. These ROS inflict oxidative damage on the DNA of bystander epithelial cells, resulting in mutations, especially mutations in tumor suppressor genes and oncogenes, that result in cellular transformation [128]. In addition to inflicting potentially mutagenic oxidative damage on the purine bases of DNA, phagocyte-derived ROS drive carcinogenesis by several other mechanisms, including oxidative inactivation of several types of DNA repair enzymes, a topic that has recently been reviewed in greater detail elsewhere [129].

### 8.2. HMGB1 and Tumor Progression: Immunosuppressive Mechanisms

The production of high levels of HMGB1 by tumor cells per se, as well as by infiltrating inflammatory cells, favors the establishment of a highly immunosuppressive TME conducive to tumor cell proliferation and progression [98,101,130]. Notwithstanding the immunosuppressive effects of sustained, inflammation-related oxidative stress, the following subsections describe additional mechanisms of HMGB1-mediated immunosuppression driving tumor progression.

#### 8.2.1. HMGB1/CXCL12/CXCR4 Axis-Driven Influx of Myeloid Suppressor Cells into the TME

Data, mostly from murine models of experimental tumorigenesis, have revealed the involvement of the HMGB1/CXCL12/CXCR4 axis in promoting the influx of immunosuppressive neutrophils, monocytes/macrophages and immature DCs, all of which have the potential to undergo transition to MDSCs in the TME [62,131,132]. In the TME, these cells encounter a cytokine milieu endowed with high levels of the immunosuppressive cytokine, transforming growth factor β1 (TGFβ1), which promotes the transition of these cells to a suppressive phenotype [133]. Largely via production of IL-10, these MDSCs not only suppress the reactivity of antitumor CD4^+^ and CD8^+^ T cells, but also decrease the expression of the naïve T cell homing receptor, L-selectin [108,131], which may underpin the exclusion of T cells from the TME, resulting in the failure of immunotherapy recently reported in patients with NSCLC [134]. In the case of TME-infiltrating immature DCs, the immunosuppressive potential of these cells is likely to be augmented via the interaction of HMGB1 with the negative immune checkpoint TIM-3 expressed on these cells [11].

Furthermore, in the clinical setting of HMGB1-related immunosuppression, immunohistochemical analysis of biopsy specimens from patients with HCC and cirrhosis (n=149), revealed that those patients with the most aggressive stage of disease and lowest survival rates (n = 59) exhibited significantly higher levels of peritumoral expression of HMGB1 and a greater influx of tumor-associated macrophages (TAMs) [135]. The authors concluded that HMGB1-mediated peritumoral influx of TAMs was indicative of an unfavorable prognosis linked to immunosuppression [135].

#### 8.2.2. HMGB1-Mediated Recruitment and Activation of Tregs

HMGB1, in various models of experimental tumorigenesis, appears to trigger the recruitment of Tregs to the TME, where these cells undergo maturation and activation, augmenting an already threatening immunosuppressive milieu [130]. While this may result from exposure of immature Tregs in the TME to IL-10 derived from various types of MDSCs [62], other mechanisms are also involved. In this context, HMGB1 apparently induces tumor cell production of thymic stromal cell lymphopoietin (TSLP), an epithelial-derived cytokine that promotes T cell maturation via interaction with antigen-presenting cells [130]. Although the data are preliminary, tumor cell-derived HMGB1 and TSLP seemingly act in concert with DCs in the TME to promote the maturation of Tregs, albeit by mechanisms that remain to be conclusively established [130].

With respect to the relevance of Tregs as mediators of immunosuppression in the clinical setting, Pang et al. investigated the relationships between the expression of HMGB1 and biomarkers of the presence and activity of Tregs in biopsies (*n* = 100) from patients with cancer of the cervix [136]. The authors observed that the levels of expression of HMGB1 correlated significantly with both clinical cancer stage and lymph node metastasis, as well as with the presence of biomarkers of Treg activation, specifically Foxp3 and IL-10 [136].

#### 8.2.3. Regulatory B Cells

Lindner et al. described the presence of unusual subsets of B lymphocytes that were present in in biopsy specimens from patients with various types of malignancy, including breast, cervical, ovarian, colorectal and prostate cancer [137]. These unusual B cells were located in close proximity to Tregs, and their transition to immunosuppressive Bregs was dependent on exposure to IL-21 secreted by Tregs. Functional immunosuppression of infiltrating antitumor T cells was achieved by expression of the serine protease, granzyme B, by Bregs, which caused proteolytic cleavage of the TCR for antigen, as well as via secretion of IL-10 and indoleamine-2,3-dioxygenase [137]. The existence of tumor-infiltrating Bregs is now well established [138,139].

With respect to involvement of HMGB1 in reprograming B cells, immunohistochemical and transcriptomic molecular analyses of biopsy specimens from patients with esophageal squamous cell carcinoma, a type of malignancy that expresses both HMGB1 and galectin, revealed infiltration of the TME by an unusual type of naïve B lymphocyte [109]. Phenotypically, these cells were CD20^+^/HMGB1 receptor^+^ and functionally responsive to HMGB1/IgM [109]. The authors contend that these cells represent a protumoral subset of B cells that is associated with “suboptimal clinical outcomes” in this type of malignancy [109]. However, these findings, which were published as an abstract, await confirmation, as does the relationship of this subset of B cells with Bregs.

### 8.3. HMGB1 and Tumor Progression: HMGB1/RAGE/NFκB-Driven Protumorigenic Mechanisms

The involvement of HMGB1/RAGE in particular, as well as HMGB1/TLR interactions, in tumor progression in both the clinical and experimental settings is now well recognized, and may involve distinct signaling pathways in different types of malignancies. In this context, earlier studies revealed that the blockade of HMGB1/RAGE interactions resulted in the attenuation of the growth and spread of both experimentally induced and spontaneous tumors in murine models of experimental tumorigenesis [140]. The attenuation of tumor growth resulted from interference with various downstream, protumorigenic intracellular signaling pathways linked to tumor cell proliferation and growth, such as those involving NFκB in particular, as well as MAPKs and interferon regulatory factors (IRFs) [140].

In the setting of HCC, the interaction of HMGB1 with RAGE expressed on a hepatic progenitor cell (HPC) line (Huh7) resulted in cellular proliferation involving activation of NFκB that was attenuated by siRNA-mediated knockdown of RAGE [104]. More recently, Khambu et al. reported that HMGB1, via interaction with RAGE expressed on isolated, autophagy-deficient murine livers, induced proliferation of HPCs and tumor promotion [105]. Mechanistically, HMGB1 released from autophagy-deficient hepatocytes promoted inflammasome-mediated activation of transcription factor NRF2 (nuclear factor erythroid-derived-2-like2; a basic zipper leucine protein) [105]. The authors contend that “HMGB1 release is a critical mechanism in hepatic pathogenesis under autophagy conditions and leads to HPC expansion as well as tumor progression” [105].

Wang et al. recently described a HMGB1/RAGE/NFκB axis-driven immunosuppressive mechanism, distinct from those described in the preceding sections, linked to the pathogenesis of melanoma [141]. These authors observed that exposure of human primary epidermal melanocytes, as well as human and murine melanocyte/melanoma/keratinocyte cell lines, to ultraviolet radiation (UVR) resulted in the expression of the inhibitory immune checkpoint molecule, programmed death (PD) ligand-1 (PD-L1) [141]. UVR-induced expression of PD-L1 by these various cell types was dependent on the release of HMGB1 as a response to cell damage, the activation of RAGE and triggering of NFκB and IRF3 [141]. The two transcription factors, in turn, formed a heterocomplex on the promotor region of PD-L1, resulting in gene transcription and expression of the immune checkpoint [141]. The expression of PD-L1 allowed melanoma cells to evade CD8^+^ T cell-mediated cytotoxicity that was preventable by blockade of HMGB1/RAGE activation or by programmed death (PD)-1/PD-L1 blockade [141]. In addition, the expression of PD-L1 enabled the survival and proliferation of an implanted PD-L1-expressing melanoma cell line (SK-mel-28) in a murine model of experimental skin tumorigenesis that was attenuated by genetic depletion of IRF3 [141].

In addition to functioning as a ligand for RAGE and TLRs, HMGB1 has also been reported to bind to triggering receptor expressed on myeloid cells (TREM)-1 [142]. This receptor, expressed predominantly on neutrophils and monocytes/macrophages, synergizes with RAGE and TLRs to amplify inflammatory signaling involving these cells of the innate immune system [142]. In this context, chronic inflammation linked to HMGB1/TREM-1 interactions has been linked to the pathogenesis of HCC and colorectal cancer [143,144].

### 8.4. Tumor Progression Driven by Cytosolic HMGB1

Notwithstanding the various protumorigenic mechanisms involving extracellular HMGB1, this protein may also drive tumor progression when present in the tumor cell cytosol during conditions of hypoxia that prevail in the TME. In the case of pancreatic tumor primary cell cultures and cell lines, for example, cytosolic HMGB1 promotes tumor proliferation by a mechanism involving the upregulated expression of mitochondrial RAGE, the activation of the mitogen-activated protein kinase kinase (MEK)/extracellular signal-regulated kinase (ERK)/MAPK pathway and increased production of adenosine triphosphate (ATP) [145].

Using murine models of experimental tumorigenesis based on injection of a cancer cell line (Hepa 1–6), Liu et al. described another unusual mechanism of HMGB1-mediated tumor progression that also occurred under hypoxic conditions [146]. These authors demonstrated that during hypoxia, HMGB1 translocates from the nucleus to the cytosol, where it binds to DNA released from damaged mitochondria, resulting in activation of intracellular TLR9 and tumor cell proliferation [146]. Such a mechanism of tumor growth was also operative during exposure of cancer cell lines (Hepa 1–6, Huh 7) to hypoxia in vitro, and was attenuated by knockdown of either HMGB1 or TLR9 [146].

### 8.5. Angiogenesis/Invasion/Metastasis Involving the HMGB1/RAGE Axis

Abundant evidence, both clinical and experimental in origin, has implicated HMGB1/RAGE interactions in potentiating tumor invasion. In an earlier study focused on gastric carcinoma, Kuniyasu et al. observed that 7/8 gastric tumor cell lines constitutively expressed RAGE-encoding mRNAs, and that the invasive potential of one of these cell lines (MKN28) was attenuated by treatment with a RAGE-targeted antisense S-oligonucleotide [147]. In addition, immunohistochemical analysis of biopsy specimens from patients with gastric carcinoma (*n* = 96) revealed that 62 of these expressed RAGE, 90% of which were poorly differentiated adenocarcinomas, with the level of expression of RAGE correlating significantly with invasive potential [147].

A later study focusing on the molecular analysis of biopsy specimens from patients (*n* = 11) with various stages of NSCLC revealed a significant association between a high level of expression of HMGB1-encoding mRNA with advanced disease and a poor prognosis [99]. The expression of HMGB1 was also positively and significantly associated with tumor expression of mRNA encoding the pro-invasive, proteolytic enzyme, matrix metalloproteinase 9 (MMP-9), both of which correlated significantly with metastatic potential [99]. The link between HMGB1 and MMP-9 was strengthened by observations that treatment of two human HMGB1-overexpressing NSCLC cell lines (A549 and H23) with HMGB1-specific siRNA significantly decreased the level of MMP-9 mRNA expression by both cell lines, as well as their metastatic potential (cellular migration and invasiveness) [99]. The intracellular signaling pathways activated in HMGB1-treated A549 cells were those involving NFκB and phosphatidylinositol 3-kinase (PI3K)/Akt. Although HMGB1/RAGE interactions were implicated in the triggering of MMP-9 expression, somewhat surprisingly, measurement of the expression of RAGE by the two airway epithelial cell lines was not performed [99].

Other types of malignancy in which the HMGB1/RAGE/NFκB axis appears to promote invasive potential include prostate cancer [101] and HCC [146]. In the case of the former, Zhang et al. reported the high-level expression of HMGB1 in prostate cancer biopsies [101]. Further investigation of the possible involvement of HMGB1 in the pathogenesis of prostate cancer revealed that exposure of a prostate cancer cell line (PC3) to recombinant HMGB1 in vitro resulted in the epithelial-to-mesenchymal transition of these cells [101]. This transition was associated with the acquisition of a pro-invasive phenotype characterized by elevated expression of mRNAs encoding MMPs-1, -3 and -10 (but not MMPs -2, -7, -8 and -9). The treatment of PC3 cells with HMGB1- or RAGE-specific siRNAs attenuated the development of these pro-invasive events, which was seemingly consistent with the involvement of the HMGB1/RAGE/NFκB axis [101].

In the case of HCC, Chen et al. reported a similar type of study [148]. These authors observed that three different HCC cell lines spontaneously expressed increased levels of mRNAs encoding HMGB1 and RAGE, which was most striking in the HPCLM3 cell line [148]. They also observed that the exposure of this cell line to recombinant HMGB1 resulted in the activation of NFκB that was associated with increased proliferative, migratory and invasive activities in these cells, all of which were attenuated by targeting of HMGB1 and RAGE with either siRNAs or specific antibodies [148]. These observations again underscore the involvement of the HMGB1/RAGE axis in tumor progression and invasion.

In addition to the aforementioned studies, all of which have convincing mechanistic components, others based exclusively on immunohistochemical detection of HMGB1, such as in cervical cancer, have described associations between the overexpression of this protein and poor prognoses [149,150]. Moreover, a meta-analysis and systematic review encompassing 18 studies and 2249 patients described a similar association in 11 different types of cancer [151]. This aspect is covered in detail in the section below that describes the clinical utility of HMGB1 as a prognostic marker in various malignancies.

### 8.6. Other Mechanisms by Which HMGB1 Promotes Tumor Spread

Several other mechanisms exist by which HMGB1 may promote tumor invasion, including: the production of CXCL12 by cancer-associated-like fibroblasts in the liver that promote the infiltration of circulating CXCR4-expressing colorectal cancer cells, as well as various types of CXCR4-expressing MDSCs and immature DCs [152]; involvement in promoting the aggression of acute myeloid leukemia (AML) via the production of high levels of HMGB1 by AML cells, which, via interaction with TIM-3 expressed on these cells, induce the autocrine secretion of pro-angiogenic vascular endothelial growth factor (VEGF) [153]; and by promoting lymphoangiogenesis in human epithelial ovarian cancer via a mechanism, albeit one that is not entirely clear, but which seemingly involves interactions between HMGB1, TAMs and lymphatic endothelial cells, as revealed by immunohistochemical analyses of tumor biopsies [103]. With respect to potential mechanisms of angiogenesis involving HMGB1/TAM interactions, Rojas et al., albeit in a series of in vitro experiments, reported that exposure of a gastric adenocarcinoma cell line (MKN45) to a M2 macrophage-like cell line in the presence of HMGB1 resulted in a series of pro-angiogenic events [154]. These included the production of MMP-9 and VEGF by the tumor cells and macrophages, respectively [154] via the production of neutrophil extracellular traps (NETs) by TANs and MDSCs of granulocytic origin. The induction of NETosis involves mechanisms that are activated by HMGB1/TLR4 interactions and tumor-derived IL-8, respectively; NETs enhance tumor invasion via the presentation of proteolytic enzymes, such as MMP-9 and elastase [155,156]. In addition, the formation of NETs in the TME may also impede the access of TILs to tumor cells [157].

Based on immunochemical analyses of breast cancer biopsies, He et al. observed that tumor overexpression of HMGB1 was associated with enhanced blood vessel formation [158]. The mechanisms involved in these pro-angiogenic activities were probed in vitro using a breast cancer cell line, (MCF-7), stably infected with the HMGB1 gene and combined with siRNA technology and assays of protein expression and cell migration [158]. The authors observed that the expression of HMGB1 by MCF-7 cells resulted in the acquisition of a provasculogenic phenotype. This transition was associated with the activation of PI3K/Akt intracellular signaling, the expression of the transcription factor hypoxia-inducible factor 1α (HIF-1α) and the synthesis of VEGF, all of which were attenuated by siRNA targeting of HMGB1 [158]. These findings, which identify a mechanistic link between HMGB1/PI3K/Akt/HIF-1α/VEGF in the induction of tumor cell-orchestrated angiogenesis, were confirmed in a murine model of experimental tumor invasion [158].

### 8.7. HMGB1, Neuro-Inflammation and Brain Metastasis

Brain metastases are recognized as “one of the deadliest forms of tumor metastasis” [159], occurring most commonly in advanced malignancies that are associated with an intense systemic inflammatory response. These include breast and lung cancers, as well as melanoma [159,160,161,162]. In this context, the incidence of brain metastasis is estimated to be 2–10 times higher than malignancies of the primary central nervous system and carrying an ominous median survival of less than one year [159].

Mechanistic studies, primarily preclinical, have revealed the involvement of neuro-inflammation in the pathogenesis of brain metastasis [159]. Preceding key events include the presence of potentially invasive circulating tumor cells derived from the primary cancer, together with a systemic inflammatory milieu conducive to the disruption of the blood/brain barrier (BBB). In this context, a BBB that is impenetrable to metastatic cancer cells requires both the structural integrity of the vascular endothelium and the expression of endothelial surface proteins/glycoproteins that protect against inflammatory insults [159].

However, in the setting of a pro-inflammatory/prothrombotic systemic milieu, the BBB is prone to attack by inflammatory mediators that include HMGB1 and thrombin [163,164]. The damaging involvement of HMGB1 in this context is underscored by experimental studies involving stereotactic injection of either the disulfide or fully reduced recombinant isoforms of HMGB1 into the brains of rats that demonstrated significant disruption of the BBB measured by magnetic resonance imaging [165]. Immunohistochemical analyses of brain tissue also revealed intense inflammatory reactions elicited by both isoforms of HMGB1 [165].

Although many of the preclinical studies on HMGB1-mediated neuro-inflammation have focused on the pathogenesis of neurological disorders [166,167], they are also likely to be implicated in establishing an immune microenvironment in the brain that favors immunosuppression and tumor growth, possibly driven by microglia and M2-like macrophages [168,169], exacerbated by HMGB1-activated NET formation by infiltrating neutrophils [170]. Clearly, however, further research is needed to unravel the precise involvement of the HMGB1/TLR4/RAGE axis in this scenario.

**Figure 3:**
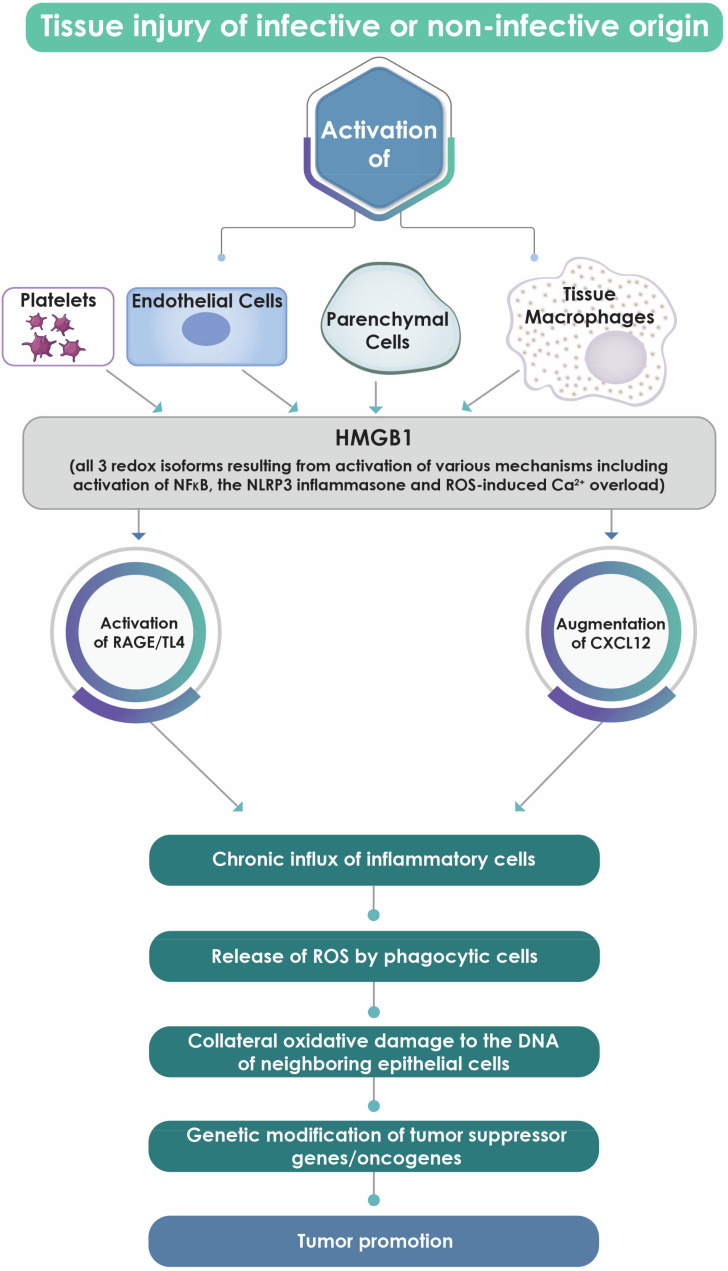
Summary of events by which HMGB1 derived from endothelial cells, tissue macrophages and parenchymal cells at sites of chronic tissue injury may drive a chronic inflammatory response that potentially leads to the development of epithelial cell injury, oxidative/inflammatory damage to DNA and tumor promotion.

**Figure 4:**
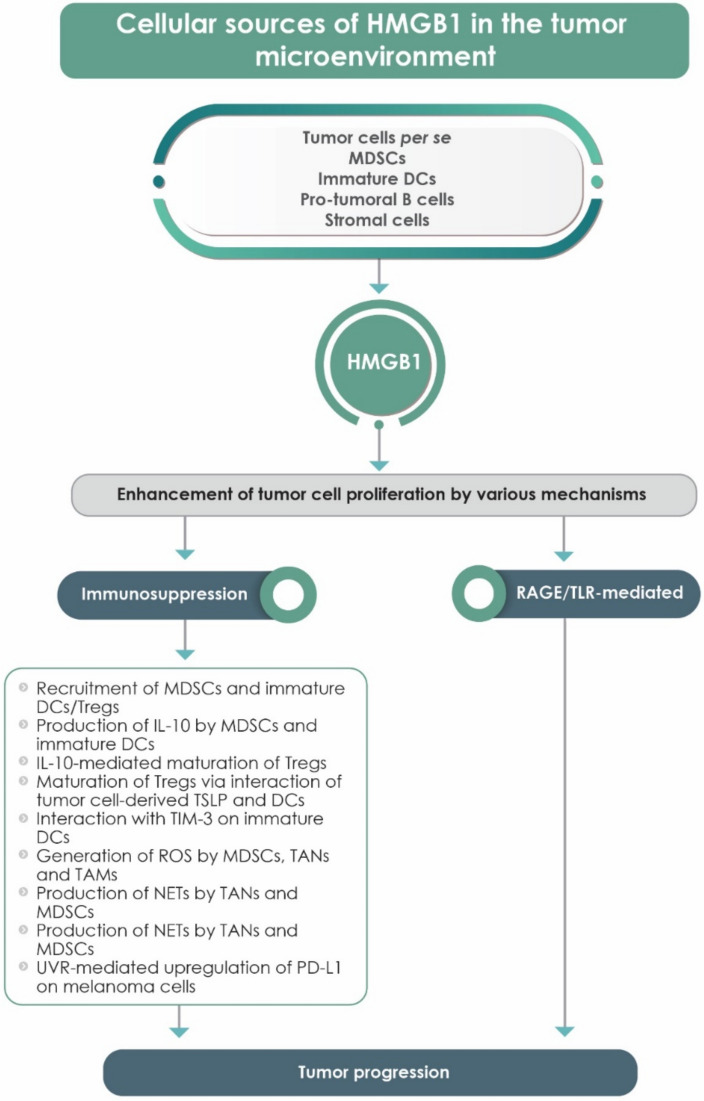
Summary of the cellular sources of HMGB1 and mechanisms of HMGB1-mediated enhancement of tumor cell proliferation. DC = Dendritic cell; IL = Interleukin; MDSC = Myeloid-derived suppressor cell; PD-L1 = Programmed death ligand 1; RAGE = Receptor for advanced glycation end products; TAM = Tumor-associated macrophage; TAN = Tumor-associated neutrophil; TIM-3 = T cell immunoglobulin mucin-3; TLR = Toll-like receptor; Treg = Regulatory T cell; TSLP = Thymic stromal cell lymphopoietin; UVR = Ultraviolet radiation.

A summary of various types of cancer and associated HMGB1-related mechanisms of tumorigenesis is shown in Table 2, while schematic representations of the putative roles of HMGB1 in inflammation/oxidative stress-mediated tumor promotion and immunosuppression-related tumor progression are shown in Figure 3 and Figure 4, respectively.

The aforementioned sections have reviewed the involvement of HMGB1 in the pathogenesis of the various stages of tumorigenesis. They clearly underscore the versatility and prominence of this protein as a master driver of these events, largely achieved via pro-inflammatory interactions with TLR4 in particular, as well as RAGE. Definitive extrapolation to the clinical cancer setting in a broader context remains somewhat difficult, however, given the experimental nature of many of the studies, as well as the different types and various stages of human cancers investigated, in addition to the various intracellular signaling pathways involved. Further clinical studies are necessary to confirm the translational relevance of these findings.

## 9. HMGB1: A Prognostic Biomarker in Cancer Patients

A number of putative cancer biomarkers have been evaluated with respect to their prognostic and/or predictive value. A prognostic biomarker offers information regarding outcome in oncological patients. Predictive biomarkers, on the other hand, help to improve treatment decisions, as they provide evidence regarding the possibility of response to a particular treatment option. Emerging prognostic biomarkers include the neutrophil/lymphocyte ratio (NLR), circulating tumor cells (CTC) or CTC products such as DNA, RNA or their protein products. Recognized predictive biomarkers include PDL-1, high tumor mutational burden and microsatellite stability [171,172,173,174,175]. Other predictive biomarkers include germline mutations in genes that encode proteins involved in the process of DNA repair, such as BRCA1 or BRCA2, or somatic mutations such as K-RAS in colon cancer or BRAF in melanoma [175,176,177]. In the case of HMGB1, the status of serum or tissue HMGB1 as a cancer biomarker is that of a prognostic biomarker at best. This contention is supported by the findings of Wu et al., who evaluated the effect of HMGB1 expression on overall survival and progression-free survival in 2249 cancer patients in a meta-analysis comprising 18 clinical studies in 11 different cancer types [151]. To our knowledge, however, no studies have identified HMGB1 as a predictive biomarker, as currently, there are no cancer treatments specifically directed against this protein.

### 9.1. Hepatocellular Carcinoma

Numerous studies have indicated that there are strong relationships between serum HMGB1 and pathological stages of HCC. HCC is a typical inflammation-related malignancy in which HMGB1 is associated with the induction of chronic inflammation, leading to an increase of extracellular matrix [178]. There is a strong correlation between levels of HMGB1 evaluated by Western blot analysis and the clinical and pathological features of HCC, including correlations with serum alpha-fetoprotein (AFP) levels and tumor size [179]. In a separate study, HMGB1 expression level also correlated inversely with tumor differentiation [180].

Experimental studies have demonstrated that HMGB1 promotes resistance to sorafenib (a targeted agent commonly used for the treatment of HCC) in HepG2 cell lines. The investigators generated HepG2 cells with HMGB1 knockdown or HMGB1 overexpression. In these experiments, HMGB1 knockdown cells showed significantly higher apoptosis and lower cell viability than normal HMGB1-expressing cells following sorafenib treatment [181]. In addition, increased tumor expression of HMGB1 correlated inversely with tumor differentiation.

In a separate study, Masuda et al. reported that high serum levels of HMGB1 predicted worse clinical prognoses in patients with HCC. In a multivariate analysis of 71 patients undergoing sorafenib treatment, these investigators identified high-level expression of HMGB1 at four weeks (*p* = 0.001), high AFP at baseline (*p* = 0.025), tumor liver occupying rate (*p* = 0.009) and modified response evaluation in solid tumors [RECIST (*p* = 0.0001)] as independent predictors of poor overall survival [182].

In keeping with these findings, a meta-analysis of 10 studies showed that HMGB1 mRNA levels in HCC were statistically significantly higher than in normal tissue (*p* < 0.00001). Overall survival was significantly shorter in HCC patients with high, compared to low, HMGB1 expression [183].

### 9.2. Non-small Cell Lung Cancer and Malignant Pleural Mesothelioma

Non-small cell lung cancer comprises different subtypes, including adenocarcinoma, squamous cell carcinoma and large cell carcinoma, that accounts for 80–85% of all lung cancers. High levels of HMGB1 have been associated with a poorer prognoses in patients with NSCLC. In this context, the expression of HMGB1 in the tissue, as well as serum levels, of patients with NSCLC were significantly higher compared to those of healthy lung tissue samples [184,185]. In a meta-analysis of 10 studies, tissue or serum levels of HMGB1 were significantly higher for NSCLC patients with stages III–IV, compared to those with stages I–II [185]. HMGB1 was also evaluated in patients with malignant pleural mesothelioma using reverse transcription-polymerase chain reaction analyses of biopsy samples. High expression levels of HMGB1 were associated with worse disease-specific survival [186].

### 9.3. Breast Cancer

In the case of Japanese breast cancer patients (*n* = 52), Aoto et al. evaluated HMGB1 using tissue immunohistochemistry [187]. Relative to pretreatment levels, they found that the expression of HMGB1 was significantly upregulated following neoadjuvant chemotherapy [187]. However, no significant correlations between HMGB1 expression and pathological response after neoadjuvant chemotherapy, or between HMGB1 expression and overall survival, were detected. Furthermore, no positive correlations between the number of CD8^+^ T cells and HMGB1 or calreticulin expression levels were evident [187].

In a separate study, the prognostic significance of plasma HMGB1, IL-6, IL-8, IL-18, MMP-2, MMP-9, glycoprotein YKL-40 and resistin were investigated in 58 metastatic breast cancer patients. In this study, only low levels of IL-8 were associated with increased overall survival, while HMGB1 was not significant [188].

### 9.4. Colorectal Cancer

Results of a meta-analysis revealed that overexpression of tissue HMGB1 correlated with clinical stage, depth of invasion, lymph node involvement and distant metastasis in Asian patients with colorectal cancer [189]. Importantly, patients with higher expression of HMGB1 had shorter overall survival relative to those patients with lower expression [189].

### 9.5. Ovarian Cancer

Lee et al. investigated the role of HMGB1 in patients with epithelial ovarian cancer using tissue microarrays of primary tumors. The study population encompassed two independent cohorts, namely a primary cohort of 194 patients and a validation cohort of 360 patients. In this study, high levels of HMGB1 were associated with an inferior progression-free survival and a trend towards overall survival [190].

### 9.6. Meta-Analysis of 11 Different Cancer Types

As mentioned, Wu et al. evaluated the effect of HMGB1 expression on overall survival and progression-free survival in 2249 cancer patients in a meta-analysis comprising 18 clinical studies in 11 different cancer types [151]. The authors studied various malignancies, including gastric, colorectal, hepatocellular, pancreatic, nasopharyngeal, head and neck squamous cell carcinoma, esophageal, malignant pleural mesothelioma, bladder, prostate, and cervical carcinomas [151]. HMGB1 overexpression was significantly associated with poorer overall survival (HR: 1.99; 95% CI, 1.71–2.31) and progression-free survival (HR: 2.26; 95% CI, 1.65–3.10), irrespective of tumor type, HMGB1 assay procedure (overexpression detected by immunohistochemistry in tissues or ELISA in serum), geographical region and study size.

In summary, numerous studies have examined the prognostic significance of HMGB1 in cancer patients, demonstrating that high levels are associated with inferior outcome, as summarized in Table 3. However, adequately designed prospective, randomized studies in specific patient populations are required to assess the prognostic utility of HMGB1 in various types of cancer.

## 10. Targeting of HMGB1 as an Anticancer Therapeutic Strategy

Various strategies to counter the pro-inflammatory (and presumably protumorigenic) activities of HMGB1 have recently been described elsewhere [191]. Amongst others, these include: (i) peptide P5779, an agent that selectively targets the HMGB1/TLR4/MD-2 pathway; (ii) an HMGB1-targeted mAb known as m2G7, which, unlike other HMGB1-targeted monoclonal antibodies, interferes with the interaction of HMGB1 with TLR4 and RAGE; and (iii) the antidiabetic agent, metformin, that inhibits the translocation of HMGB1 to the cytosol [191]. To date, P5779 and m2G7 have demonstrated anti-inflammatory activity in murine models of experimental infection, as well as in models of experimental neurological and cardiovascular disease, but, to our knowledge, these agents have not been evaluated in the setting of oncotherapy. In the case of metformin, diabetic patients treated with this agent appear to experience a decreased likelihood of developing various types of cancer [192]. In addition, the potentially beneficial effects of this agent as an adjunct to cancer treatment are being investigated in several on-going clinical trials [192]. However, it remains to be determined which of metformin’s various mechanisms of anti-inflammatory activity [192] is/are operative in cancer prevention and/or therapy.

Somewhat paradoxically, HMGB1, as mentioned above, together with several other DAMPs, is considered a key mediator of ICD activated by several different types of chemotherapeutic agents and radiotherapy [90,91,92,93,94]. However, given that HMGB1 has been reported in a number of studies to promote resistance to radiation and chemotherapy [98,193,194], revealing insights into the cellular origins and regulatory mechanisms involved in chemical modification of the protein, as well as the biological activities of the various redox- and enzymatically-modified variants of HMGB1, is a priority [195].

## 11. Conclusions

HMGB1 is a ubiquitous, multifaceted molecule that is increasingly implicated in the pathogenesis of many types of human cancer, driving all of the stages of tumorigenesis. HMGB1 is utilized by tumor cells to divert the potentially protective activities of the innate immune system, specifically those involving activation of TLR4, as well as RAGE, to generate a hyperinflammatory, immunosuppressive, protumorigenic TME. The involvement of HMGB1 in tumorigenesis is, however, complex, a contention that is underscored by the existence of numerous redox- and enzymatically-modified variants of this molecule, several with well-characterized biological activities, and others less so. Notwithstanding the importance of the acquisition of definitive insights into the origins, regulation of chemical modification and biological functions of HMGB1 variant molecules, several other key areas of the role of HMGB1 in cancer also necessitate clarification. These include defining the exact nature of the involvement of HMGB1 in ICD, either as a key mediator or as an impediment to the antitumor activities of other DAMPs released during this process. In addition, despite its prognostic potential, the clinical utility of targeting HMGB1 in anticancer immunotherapy is a priority issue that requires further research. In this latter context, extensive HMGB1 isoform studies may make it possible to distinguish between the protumorigenic and antitumorigenic activities of this protein and further define its potential role as a prognostic and predictive cancer biomarker.

## Figures and Tables

**Table 1:** Summary of effects of HMGB1 on cells of the immune system in health and disease.

Immunostimulatory Properties	
Cell Type & Activities Affected	References
Adhesion and migration of monocytes, & neutrophils	[54,57]
Activation of NFκB with release of pro-inflammatory cytokines in monocytes, macrophages, neutrophils and dendritic cells	[52,55,56,59]
Activation of NADPH oxidase and production of ROS by neutrophils and NO production by macrophages	[56,58]
Expression of MHCII on macrophages	[56]
Skew macrophages to a pro-inflammatory M1 phenotype in SLE and an experimental model of autoimmune myocarditis	[60,61]
Maturation of dendritic cells that drives Th1 polarization	[52,53,68]
Migration and accumulation of dendritic cells	[66,67,68]
Crucial role in NK development, IL-2 induced proliferation, NK bioenergetics, and diverse NK functions including tumor control	[80]
Proliferation of CD4+ and CD8+ T cells	[70]
The expression levels of CTLA-4 and Foxp3, both essential for their immunosuppressive functions, including IL-10 secretion, were found to be diminished in T regulatory cells	[71,72,73]
**Immunosuppressive Properties**	
Promote differentiation and proliferation, as well as suppressive activity of myeloid-derived suppressor cells	[62,63]
In contrast to above, increased suppressive function and prolonged survival of T regulatory cells	[74]

**Table 2:** Some human cancers in which HMGB1 has been implicated in disease pathogenesis.

Type of Malignancy	Reported Involvement of HMGB1 in Pathogenesis	References
Non-small cell lung cancer	Involvement of the HMGB1/RAGE/NFκB axis in promoting tumor invasion via production of tumor cell MMP-9	[99]
Metastatic pancreatic ductal adenocarcinoma	Involvement of the HMGB1/RAGE/NFκB axis in promoting epithelial-to-mesenchymal transition and production of MMPs -1, -3, -10, resulting in a pro-invasive phenotype	[100,101]
Metastatic breast cancer	Increased tumor expression of HMGB1 correlates with tumor stage and metastatic potential	[21,102]
Epithelial ovarian cancer	Interactions between HMGB1, TAMs* and lymphatic endothelial cells promote endothelial cell proliferation and lymphangiogenesis	[103]
Hepatocellular carcinoma	Involvement of the HMGB1/RAGE/NFκB axis in promoting tumor cell proliferation and acquisition of an invasive phenotype	[104,105,135,143]
Colorectal cancer	Involvement of HMGB1 in tumor progression via recruitment of MDSC, and metastasis via CXCR4/CXCL12-driven mechanisms	[106,107,133,152]
Metastatic melanoma	Immunosuppression driven by the HMGB1/RAGE/NFκB axis involving production of IL-10 by M2-like macrophages in the TME and expression of PD-L1 on tumor cells	[108,141]
Esophageal squamous cell carcinoma	Infiltration of the TME by immature pro-tumoral B-lymphocytes	[109]
Malignant mesothelioma	HMGB1 promotes tumor growth, migration and invasion by mechanisms that remain to be identified, but are possibly pro-inflammatory/immunosuppressive in nature	[110]
Glioblastoma	Promotes tumor growth and spread by pro-inflammatory mechanisms associated with vascular leakage and edema in the TME	[111]
Acute myeloid leukemia (AML)	Interacts with the immune checkpoint, TIM-3, expressed on human AML cells to induce autocrine production of pro-angiogenic VEGF and disease progression	[153]
Metastatic gastric cancer	HMGB1/RAGE strongly correlated with tumor invasive potential	[147]

**Table 3:** Human cancers in which HMGB1 has been implicated in disease prognosis.

Type of Malignancy	References
Hepatocellular carcinoma	[178,179,180,181,182]
Non–small cell lung cancer	[184,185]
Malignant pleural mesothelioma	[186]
Breast cancer	[187,188]
Colorectal cancer	[189]
Ovarian cancer	[190]
Meta-analysis of 11 different cancers	[151]

## Abbreviations

MMP	matrix metalloproteinase
TAM	tumor-associated macrophage
TME	tumor microenvironment
VEGF	vascular endothelial growth factor
